# Capacity Development of Local Service Organizations Through Regional Innovation in Papua, Indonesia After the COVID-19 Pandemic

**DOI:** 10.3389/fpsyg.2022.912692

**Published:** 2022-05-30

**Authors:** Andjar Prasetyo, Dewi Gartika, Agustinus Hartopo, Bekti Putri Harwijayanti, Sukamsi Sukamsi, M. Fahlevi

**Affiliations:** ^1^Regional Development Planning Agency of Magelang City, Semarang, Indonesia; ^2^Research and Development Agency of West Java Province, Bandung, Indonesia; ^3^Regional Development Planning Agency of Papua Province, Jayapura, Indonesia; ^4^Poltekkes Kemenkes Semarang, Semarang, Indonesia; ^5^Regional Development Planning Agency of Magelang Regency, Semarang, Indonesia; ^6^Management Department, Bina Nusantara University and Priviet Research Lab, Jakarta, Indonesia

**Keywords:** local service, organizations, regional, capacity development, innovation

## Abstract

This study aims to identify and describe the regional innovations produced in Keerom Regency, Papua Province, Indonesia after the Coronavirus disease 2019 (COVID-19) pandemic, to analyze integrated regional service capacity indicators with a special focus on organizational performance indicators in integrated units that can be measured quantitatively and simply. In addition, to create an understanding of organizational performance in geographic areas. The method approach uses a mixed-methods description to tell the results of the study. Secondary data were analyzed in the form of innovation proposals for as many as 108 regional innovations. The analysis used adopts local service capacity which was developed with additional indicators of innovation. Local Service Indicators used to consist of Leadership and Governance, Structure and System, Human Resources, Financial Management, Program Management, Monitoring and Evaluation Plan, Partnerships, External Relations and Networks, Knowledge Management, and Organizational Innovation. Local services are built using spreadsheet tools to make the process easier to use. The result is that organizational services in regional innovation as a whole still reach a score of 52% on a 100% scale giving the result that organizational services have been carried out amid the limitations of local governments which are characterized by limited knowledge needed, not yet optimally prepared programs, low organizational acceleration. For regional innovation, the harmonization process between planning, evaluation, and monitoring is not yet optimal.

## Introduction

The performance of the organizational structure has changed massively and globally since the Coronavirus disease 2019 (COVID-19) pandemic, including in Indonesia. Indonesia has the character of a government organization with a tiered structure from National, Province to Regency/City which is dynamic due to global changes. The organizational performance approach generally forms a different character from one region to another; meanwhile to encourage the creation of organizational performance quantitative parameters that can be measured have not emerged. Inequality dimensions, such as time, human resources, location, and organizational culture have an impact on differences in organizational performance results. Areas near the center of the national government develop faster than those located far from the center of the national government in terms of organizational performance. Some literacy has contributed to organizational performance, but is still done partially, from the perspective of Leadership and Governance; Structure and System; Human Resources; Financial Management; Program Management; Monitoring and Evaluation Plan; Partnerships, External Relations, and Networks; Knowledge Management; and Organizational Innovation. Leadership and Governance tagged with articles from Ansell et al. (2021), Areal and Sheppy (2021), and Argaw et al. (2021).

Next on Human Resources, which has been discussed by Bonache and Festing (2020), Carnevale and Hatak (2020), and Azizi et al. (2021). The next discussion of organizational performance is Financial Management, as described by Hilimi et al. (2020), Kaunang (2020), and Basri et al. (2021). Program Management Plans are also mentioned as part of organizational performance, with scientific literacy being discussed by Adjekum and Tous (2020), Rahayu (2020), and Rodríguez-Fernández et al. (2021). The organizational performance also has a sustainability impact which is marked by the Monitoring and Evaluation process, with scientific literacy constructed (Masawe and Isanzu, 2020; Palmer et al., 2020; Podolyanchuk, 2020). Partnerships being part of accelerating organizational performance have received scientific attention referring to the article (Franco and Haase, 2020).

The growth of organizational performance requires interaction in the form of External Relations and Networks, it has been mentioned in several scientific articles, for example, adopting from Aminullah et al. (2020). Capacity and capability in organizational performance are also influenced by how the pattern and construction of Knowledge Management are described (Ao and Liu, 2013; Maligat et al., 2020). Organizational innovation has an important role in organizational performance, meanwhile, literacy support that raises this issue can be referred to Alfawaire and Atan (2021). Organizational innovation is believed to be a component that contributes to improving organizational structures that lead to increased performance capacity that adopts any action that increases the effectiveness of individuals, organizations, networks, or systems—such as organizational and financial stability, program service delivery, program quality, and growth.

Organizational performance has an important role in increasing the maturity of local government in Indonesia. One of the areas far from the center of government in Indonesia is the Keerom Regency, which is a border area with the state of Papua New Guinea and part of the Papua Province of Indonesia. The position of this region is interesting for a study because the study locus has the capability of committed innovation management human resources from the leader level to implementers in organizations in the Regional Government; another consideration is seen on a national scale in 2021. Keerom Regency has the potential for innovation that contributes to the development innovation in Papua Province and Nationally. But still do not fully understand how the organization’s services are implemented in the management of regional innovation.

This article builds a model for the organizational performance gaps with a service approach, as an empirical contribution by measuring the organizational performance. Empirical evidence was carried out in Keerom Regency as an area far from the center of the national government. Empirical research on organizational performance is very important considering the current and future challenges to increase equitable development and reduce disparities between regions. From a regional perspective, this research contributes theoretical insights regarding the same organizational performance indicators despite differences in time, human resources, location, and organizational culture. This study aims to identify and describe the regional innovations produced, and to analyze integrated regional service capacity indicators with a special focus on organizational performance indicators in integrated units that can be measured quantitatively and simply. In addition, to create an understanding of organizational performance concerning geographic areas.

## Methods

**FIGURE 1:**
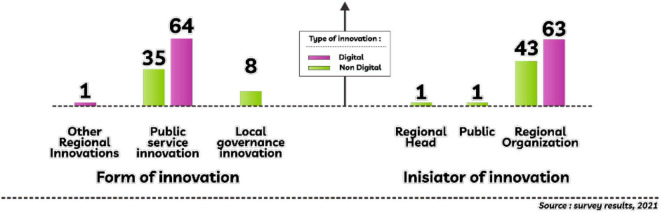
Form, type, initiator of innovations in Keerom Regency.

The approach method uses a description of the mixed methods to narrate the results of the study. To support the analysis used secondary data obtained from the results of online interactions with the Keerom Regency Government, secondary data were analyzed in the form of innovation proposals for as many as 108 regional innovations. The sample in this study is the result of the identification of regional innovation proposals. The innovation proposals came from 35 regional apparatus organizations in the Keerom Regency Government, Papua Province, Indonesia as well as being the locus of this research for 6 months from June to November 2021. Data were obtained through interviews and discussions with stakeholders shown in Figure 1. Full identification and interviews through mobile and internet-based communication channels in obtaining secondary data and primary data. The analysis used adopts local service capacity which was developed with additional indicators of innovation. The local Service Indicators used to consist of (1) Leadership and Governance; (2) Structure and System; (3) Human Resources; (4) Financial Management; (5) Program Management; (6) Monitoring and Evaluation Plan; (7) Partnerships, External Relations, and Networks; (8) Knowledge Management; and (9) Organizational Innovation. Local services are built using spreadsheet tools to provide convenience in the utilization process. An analysis is measured using a Likert scale to translate quality into quantity with a value of one to five. Respondents’ answers are described in no activity is given a value of one; minimum activity is given a value of two; moderate activity is given a value of three; significant activity is given a value of four; and a value of five for optimal activity.

## Results and Discussion

### Keerom Regency Innovation

The Keerom Regency is one part of the expansion area of Jayapura Regency. The regional expansion has been carried out since 2002 based on the Law Number 26 of 2002 concerning the Establishment of Sarmi Regency, Keerom Regency, South Sorong Regency, Raja Ampat Regency, Bintang Mountains Regency, Yahukimo Regency, Tolikara Regency, Waropen Regency, Kaimana Regency, Bouven Digul Regency, Asmat Regency, Mappi Regency, Teluk Wondama Regency, Teluk Bintuni Regency, in Papua Province (State Gazette Year 2002 Number 129, Supplement to State Gazette Number 4,245). Geographically, the Keerom Regency is located along the border area of the Republic of Indonesia with the State of Papua New Guinea (PNG), Astronomically, it is located at 14 00 15’–14 10’ 0’ east longitude and 20 370”–40 0’0” south latitude. Administratively, the administrative boundaries of Keerom Regency are in the north, bordering Jayapura City and Jayapura Regency; in the south bordering the Bintang Mountains Regency; the west is bordered by Jayapura Regency; and the east is bordered by the State of PNG. The district capital, located in Arso District, has a direct impact on the ease with which areas in this district can access the center of government. Keerom Regency is divided into 11 district administrations, from the 11 existing districts, Senggi District which is on the southwest side is the district with the widest area of 2,538.00 Km^2^ or 27.10 percent of the total area of Keerom Regency. Meanwhile, Mannem is the district with the smallest area of 160.36 km^2^ or only 1.71 percent.

In the regulation, it is stated that the regional innovation consists of three forms, namely governance, public services, and other affairs, for the form of innovation from the survey results obtained as many as eight innovations in governance, one innovation in another affairs and 99 forms of innovation in public services. Furthermore, the initiators are divided into several, starting from the regional head producing one regional innovation, the community getting one regional innovation, and local government organizations dominantly being able to produce as many as 106 regional innovations.

Then referring to the Law Number 23 of 2014 concerning Regional Government, it is stated that the several affairs are handled by regional governments. These affairs are also used as a differentiator in government affairs for regional innovation.

**FIGURE 2:**
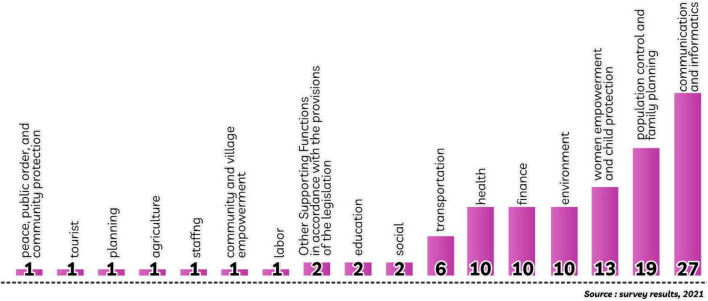
Government affairs of innovations in Keerom Regency.

The results of the identification of regional innovations based on regional government affairs as shown in Figure 2. For (1) affairs of peace, public, and community protection; (2) tourism affairs; (3) planning affairs; (4) agricultural affairs; (5) personnel affairs; (6) the affairs of community and village empowerment; (7) the work affairs of each as much as one regional innovation. Furthermore, for (1) the affairs of other supporting functions by the provisions of the legislation; (2) educational affairs; and (3) social affairs with two regional innovations each. Then as many as six regional innovations as affairs were included in transportation. On the (1) health affairs; (2) financial affairs; and (3) environmental affairs with ten regional innovations each. Thirteen innovations become data in affairs of women’s empowerment and child protection, followed by more control and family control is the development of nineteen regional innovations. Finally, as many as twenty-seven regional innovations became affairs of communication and informatics. These results indicate that there are differences in quantity between affairs, even though they have the same capacity and ability according to their respective affairs. This is indicated by the service organization services between affairs which are allegedly due to the unequal availability of the number of organizational service indicators that are weak in the quality of regional innovation. In the next discussion, the analysis of organizational services, and the organization of the interview process through telephone communication and other relevant media.

### Organizational Performance Indicators

The results of the identification of 108 regional innovations were then measured by the local Service Indicators used, consisting of the first indicator being Leadership and Governance, being a determining indicator in organizational services, in line with the article (Argaw et al., 2021; Kusmiarto et al., 2021; Mensah, 2021; Taryono et al., 2021), this indicator is limited to five components consisting of (1.1) The vision and mission of the organization; (1.2) The strategic plan; (1.3) Harmonization of regional strategic plans with Provincial/National Strategic Plans; (1.4) Organizational structures; (1.5) Organizational functions and organizational governance. Then the second indicator Structure and System, which is the main key in organizational service, is in line with the study (Smith et al., 2012; Srinivasan et al., 2019; Flores-Cabezas et al., 2020). This indicator has at least six components consisting of (2.1) Organizational policies and procedures; (2.2) Organizational steps; (2.3) Consultation and decision making; (2.4) Internal communication; (2.5) Accountability and transparency; and (2.6) Office and equipment. Furthermore, the third indicator, Human Resources, is an important indicator that can run the organization’s services technically. Scientific articles that are in line with this indicator as mentioned (Shrouf et al., 2020; Gonçalves et al., 2021; Murawski, 2021). This indicator is indicated by eight components which are detailed in (3.1) Recruitment, staff diversity, and expertise; (3.2) Roles, responsibilities, and job descriptions; (3.3) Personnel files; (3.4) Timesheets; (3.5) Volunteer/Internal management; (3.6) Discipline, grievances, and conflict resolution; (3.7) Staff performance evaluation; and (3.8) Development staff.

The fourth indicator of the Financial Management, it has a supporting role that contributes to the smooth running of the organization’s service processes. This indicator has been widely discussed in several scientific references, for example by Mardiana (2021) and Maswadeh and Al Zumot (2021). This indicator has 12 components, the details are as follows (4.1) Financial accounts/accounting system; (4.2) Financial record keeping; (4.3) Budgets and cash flow planning; (4.4) Cost-effectiveness; (4.5) Finance staff levels and competency; (4.6) Financial reporting; (4.7) Financial compliance to statutory regulations; and (4.8) Financial policies.

The next indicator, namely the fifth is Program Management, this indicator is oriented to the form of organizational services that will be implemented, and has scientific literacy that is in line with Adjekum and Tous (2020) and Wang and Wu (2020) components of this indicator can be developed, but this study contains six components consisting of (5.1) Innovation information, knowledge, and skills; (5.2) Program design and modification; (5.3) Program implementation review; (5.4) Service delivery; (5.5) Sustainability program; and (5.6) Resource mobilization and sustainability.

The sixth indicator, Monitoring and Evaluation becomes an indicator of organizational services used after the implementation of organizational services and strengthens the articles discussed (Astuti, 2020; Hutomo et al., 2020; Putra et al., 2020). This indicator consists of five components including (6.1) Annual work plans; (6.2) M&E plans and frameworks; (6.3) M&E tools and data collection system; (6.4) M&E data analysis, dissemination, and use; and (6.5) Evaluation contributes to organizational learning.

The seventh indicator is Partnerships, External Relations, and Networks, being an accelerator in organizational services and line with studies (Clough and Piezunka, 2020; Rienties and Hosein, 2020; Capodistrias et al., 2021; de Almeida et al., 2021). This indicator has at least four components which can be detailed as follows (7.1) Community presence and involvement; (7.2) External communication strategy; (7.3) Communication materials; and (7.4) Advocacy and policy engagement.

The eighth indicator is Knowledge Management, shaping the distribution of knowledge that can be adapted and implemented to the service structure and adopts deep thinking (Maligat et al., 2020). This indicator includes two components, namely (8.1) Knowledge exchange; (8.2) Knowledge management; (8.3) Knowledge maturity; and (8.4) Knowledge dynamics.

The last indicator is organizational innovation as an indicator that shows the required service activities, referring to the study by Lambert and Lapsley (2010), Mensah (2021), and Susiatiningsih et al. (2021) covering six components consisting of (9.1) innovation regulation; (9.2) innovation resources; (9.3) infrastructure, facilities, and work methods; (9.4) innovation culture; (9.5) information or documentation systems; and (9.6) results of strengthening innovation.

These nine indicators can be used as benchmarks for organizational performance, not limited to regional innovation, but can be developed for other fields. The resulting regional innovation portrait will be better if it has the required components in the organizational performance indicators.

## Discussion

The nine indicators that have been detailed in the 52 components are then quantified on a spreadsheet scale to analyze the services of regional innovation organizations. The analysis is limited to interactions that have been carried out with regional apparatus organizations in the Keerom Regency, then interpreted with a subjective scale on each component and then summarized in each indicator qualitatively. The higher the quantitative results or closer to the baseline, the more fulfilled the organization’s service indicators are, while the measurement results are presented in Table 1.

**TABLE 1:** Results of measuring organizational services in regional innovation.

No.	Indicator	Component	Respond	Results	Baseline
1	Leadership and governance	1.1. The vision and mission of the organization	Optimal activity	5	5
		1.2. The strategic plan	Significant activity	4	5
		1.3. Harmonization of regional strategic plans with Provincial/National Strategic Plans	Significant activity	4	5
		1.4. Organizational structure	Significant activity	4	5
		1.5. Organizational functions and organizational governance	Significant activity	4	5
		Total		21	25
2	Structure and system	2.1. Organizational policies and procedures	Moderate activity	3	5
		2.2. Organizational step	Minimum activity	2	5
		2.3. Consultation and decision making	Moderate activity	3	5
		2.4. Internal communication	Significant activity	4	5
		2.5. Accountability and transparency	Moderate activity	3	5
		2.6. Office and equipment	Moderate activity	3	5
		Total		18	30
3	Human resources	3.1. Recruitment, staff diversity, and expertise	Moderate activity	3	5
		3.2. Roles, responsibilities, and job descriptions	Significant activity	4	5
		3.3. Personnel files	Moderate activity	3	5
		3.4. Timesheets	Minimum activity	2	5
		3.5. Volunteer/Intern management	Minimum activity	2	5
		3.6. Discipline, grievance, and conflict resolution	Moderate activity	3	5
		3.7. Staff performance evaluation	Minimum activity	2	5
		3.8. Staff development	Significant activity	4	5
		Total		23	40
4	Financial management	4.1 Financial accounts/ accounting system	Moderate activity	3	5
		4.2. Financial record-keeping	Moderate activity	3	5
		4.3. Budgets and cash flow planning	Minimum activity	2	5
		4.4. Cost-effectiveness	Minimum activity	2	5
		4.5. Finance staff levels and competency	Moderate activity	3	5
		4.6. Financial reporting	Significant activity	4	5
		4.7. Financial compliance with statutory regulations	Significant activity	4	5
		4.8. Financial policies	Optimal activity	5	5
		Total		26	40
5	Program management	5.1. Innovation information, knowledge, and skills	No activity	1	5
		5.2. Program design and modification	Minimum activity	2	5
		5.3 Program implementation review	Moderate activity	3	5
		5.4 Service delivery	Optimal activity	5	5
		5.5 Program sustainability	No activity	1	5
		5.6 Resource mobilization and sustainability	No activity	1	5
		Total		13	30
6	Monitoring and evaluation plan	6.1. Annual work plan	Significant activity	4	5
		6.2. M&E plans and framework	Moderate activity	3	5
		6.3. M&E tools and data collection system	Minimum activity	2	5
		6.4. M&E data analysis, dissemination, and use	Minimum activity	2	5
		6.5. Evaluation contributes to organizational learning	Minimum activity	2	5
		Total		13	25
7	Partnerships, external relations, and networks	7.1. Community presence and involvement	Significant activity	4	5
		7.2. External communication strategy	Optimal activity	5	5
		7.3. Communication materials	Significant activity	4	5
		7.4 Advocacy and policy engagement	Significant activity	4	5
		Total		17	20
8	Knowledge management	8.1. Knowledge exchange	Minimum activity	2	5
		8.2. Knowledge management	Minimum activity	2	5
		8.3. Knowledge maturity	Minimum activity	2	5
		8.4. Knowledge dynamic	Minimum activity	2	5
		Total		4	20
9	Organizational innovation	9.1. Innovation regulation	No activity	1	5
		9.2. Innovation resources	Moderate activity	3	5
		9.3. Infrastructure, facilities, and work methods	Minimum activity	2	5
		9.4. Innovation culture	No activity	1	5
		9.5. Information or documentation systems	Moderate activity	3	5
		9.6 Results of strengthening innovation	Minimum activity	2	5
		Total		12	30
		Grand total		147	260

*Analysis Primary data, 2022.*

The total results of the service analysis scored 147 points from a baseline of 260 points, derived from (1) Leadership and Governance of 21 points from a baseline of 35 points, this shows that Leadership and Governance are in regional innovation but still need to be improved, especially in the distribution of governance; (2) Structure and System has a score of eighteen points from a baseline of thirty points explaining that organizational services in regional innovation as a whole do not have system/structure maturity even though internal communication has been well developed. (3) The Human Resources indicator obtained a score of 23 points from a total baseline of forty points, which means that organizational services in regional innovation are still low, which is indicated by the undeveloped capacity and capability of human resources in handling regional innovation. (4) Financial management scored 26 points from a baseline of fourth points, indicating that the ability of the budget for organizational services in regional innovation has been well planned but is still partial in its implementation, the need for efforts to encourage clear financial availability in the development of regional innovation. (5) Program Management obtained a score of thirteen points from a baseline of thirty points indicating that the organization’s services for regional innovation have not been programmed properly and maturely, so they still do not know the claims of regional innovations that have an impact on organizational services. (6) The Monitoring and Evaluation Plan received a score of thirteen points from a baseline of 25 points, assuming that although it has an organizational service plan for the development of regional innovation, monitoring, and evaluation of the quality and quantity of the organization’s services has not been carried out. (7) Partnerships, external relations, and networks scored seventeen points on a scale of 20 points, it can be assumed that organizational services in regional innovation have been strived to develop by involving stakeholders who have the ability and capacity to encourage the achievement of quality regional innovation. (8) Knowledge management gets a score of four points out of a twenty-point scale, ideally, this organization’s services have dynamic sustainability in the implementation of regional innovations, even though in existing conditions this indicator occupies the lowest position compared to other indicators. (9) Organizational innovation, getting a score of 12 points from a baseline of thirty points, provides a description that organizational services in regional innovation do not yet have legal force because local government regulations that legitimize regional innovation do not yet exist, even though the source and process for documenting organizational services in regional innovation have been carried out.

## Conclusion

The Keerom Regency has the potential for regional innovation which is indicated by the number identified, but it has not been integrated into organizational performance. Organizational services in regional innovation as a whole still reach a score of 52% on a scale of 100% giving the result that organizational services have been carried out in the midst of the limitations of local governments which are characterized by limited knowledge required, not yet maximally prepared programs, low organizational acceleration for regional innovation, not yet optimal, optimizing the harmonization process between planning evaluation and monitoring. However, the commitment to Leadership and Governance has been recognized as the basis for improving organizational services in regional innovation, which is supported by the readiness of structures and systems to encourage organizational service quality in regional innovation. In addition, the support of human resources and financial capabilities complemented by more mature partnerships, external relations, and networks is believed to be able to increase the acceleration of organizational services in regional innovation.

Thus, regional services with regional innovation development indicators can be used as an option in the process of achieving organizational targets, in addition to contributing to enriching regional organizational development tools and regional innovations. The analytical tool used is an alternative in contributing to improving organizational performance not only on regional innovation but can be implemented in other ways in local government, by adjusting the character understudy to be a limitation in this study.

## Author Contributions

All authors listed have made a substantial, direct, and intellectual contribution to the work, and approved it for publication.

## Conflict of Interest

The authors declare that the research was conducted in the absence of any commercial or financial relationships that could be construed as a potential conflict of interest.

## Publisher’s Note

All claims expressed in this article are solely those of the authors and do not necessarily represent those of their affiliated organizations, or those of the publisher, the editors and the reviewers. Any product that may be evaluated in this article, or claim that may be made by its manufacturer, is not guaranteed or endorsed by the publisher.
